# Policy exclusion or confusion? Perspectives on universal health coverage for migrants and refugees in South Africa

**DOI:** 10.1093/heapol/czab038

**Published:** 2021-04-13

**Authors:** Janine A White, Laetitia C Rispel

**Affiliations:** School of Public Health, Faculty of Health Sciences, University of the Witwatersrand, Johannesburg, 27 St Andrew’s Road, Parktown, Johannesburg 2193, South Africa; Centre for Health Policy & South African Research Chair, School of Public Health, Faculty of Health Sciences, University of the Witwatersrand, Johannesburg, 27 St Andrew’s Road, Parktown, Johannesburg 2193, South Africa

**Keywords:** Universal health coverage, migrants, NHI, South Africa, social exclusion

## Abstract

Notwithstanding the promise of the inclusivity of universal health coverage (UHC), the integration of migrants and refugees into host countries’ health systems remains elusive and contested. In South Africa, there is insufficient scholarly attention on UHC, migrants and refugees, given the country’s strategic importance in Africa and the envisaged implementation of the National Health Insurance (NHI) system. In this paper, a social exclusion conceptual framework is used to explore whether South African legislation, health policies and perspectives or actions of health policy actors facilitate UHC for migrants and refugees or exacerbate their exclusion. We combined a review of legislation and policies since 1994, with semi-structured interviews with 18 key informants from government, academia, civil society organizations and a United Nations organization. We used thematic analysis to identify themes and sub-themes from the qualitative data.

The South African Constitution and the National Health Act facilitate UHC, while the Immigration Act and the 2019 NHI Bill make the legal status of migrants the most significant determinant of healthcare access. This legislative disjuncture is exacerbated by variations in content, interpretation and/or implementation of policies at the provincial level. Resource constraints in the public health sector contribute to the perceived dysfunctionality of the public healthcare system, which affects the financial classification, quality of care and access for all public sector patients. However, migrants and refugees bear the brunt of the reported dysfunctionality, in addition to experiences of medical xenophobia. These issues need to be addressed to ensure that South Africa’s quest for UHC expressed through the NHI system is realized.

**Key messages**

- A social exclusion conceptual framework is a useful analytical tool to explore universal healthcare for migrants and refugees, by focusing attention on the structural drivers of exclusion, the processes that generate unequal power relationships, the policy actors that drive exclusion and the intersection among all of these.
- Despite a right-based and non-discriminatory constitution and an enabling National Health Act in South Africa, both the Immigration Act and the 2019 NIH Bill make legal status of migrants the most significant determinant of their healthcare access.
- The intersection of under-investment, resource constraints and perceived dysfunctionality of the public health sector contribute to the exclusionary behaviour and actions of frontline health workers against migrants and refugees.
- The reported discrimination and stigma experienced by migrants and refugees include the request for identity documents, financial misclassification, discretionary healthcare access, denial of treatment, and in some instances name-calling and outright discrimination by frontline health workers.

## Introduction

Universal health coverage (UHC) is a key target of the United Nations’ Sustainable Development Goals, contributing to the reduction of health inequities and improvements in population health outcomes (United Nations, 2019, p. 6). UHC means that all people receive the quality health services they need, while ensuring that health service utilization does not expose users to financial hardship (WHO, 2019, p. 7). UHC embodies the concepts of equity, quality and financial risk protection (WHO, n.d.) and is intended to ‘leave no one behind’ (WHO, 2019, p. 7). This inclusive nature of UHC was captured in the political declaration of the 2019 high-level meeting on UHC (United Nations, 2019) and implies the prioritization of vulnerable groups, such as migrants (WHO, 2019; United Nations, 2019). However, achieving UHC is complex, influenced by country context and different meanings of the concept. UHC implementation requires clarity on prioritization, regulatory frameworks, financing mechanisms and potential trade-offs given resource constraints in many countries (Jha *et al.*, 2016; Norheim, 2016; Rispel, 2019; Rumbold *et al.*, 2017).

The 2018 Lancet Commission on Migration underscored the responsibility of governments to uphold human rights and provide equitable UHC to migrant populations, regardless of their legal status (Abubakar *et al.*, 2018). Despite the promise of the inclusivity of UHC, the integration of migrants and refugees into host countries’ health systems remains elusive and contested (Wickramage *et al.*, 2018; Legido-Quigley *et al.*, 2019). In this study, we refer to migrants as individuals who have moved across an international border away from their place of residence, regardless of their legal status, the voluntary nature of movement and/or the causes for the movement (International Organization for Migration, 2019). The United Nations defined a refugee as ‘someone who is unable or unwilling to return to their country of origin owing to a well-founded fear of being persecuted for reasons of race, religion, nationality, membership of a particular social group, or political opinion’ (United Nations, 1951, p. 3). In practice, a refugee refers to an individual with formal documentation who has been granted refugee status by the host country.

In recognition of the social exclusion of migrants and refugees from health policies, the 2019 World Health Assembly agreed that one of the key priorities in all Member States should be to mainstream migrant and refugee health into health policies (World Health Organization, 2019). However, research studies have documented the entire spectrum of health policies in countries, from those that are inclusive of all migrants regardless of their legal status, to much more restrictive policies, eroding the principles of UHC (Legido-Quigley *et al.*, 2019; Cabieses *et al.*, 2019; Yaya and Sanogo, 2019).

In the European Union (EU), several studies have found considerable variation in healthcare access for migrants and refugees, between and within EU Member States (Cuadra, 2011; Woodward *et al.*, 2013; De Vito *et al.*, 2015; Keith and Van Ginneken, 2015; Cimas *et al.*, 2016; Hannigan *et al.*, 2016; Geeraert, 2018; Ledoux *et al.*, 2018). A 2016 review of countries in the EU found that legal status was one of the most significant factors that influenced migrants’ access to comprehensive health services (Hannigan *et al.*, 2016). Within the EU, undocumented migrants mostly had access to emergency care, but both formal and informal barriers hindered access in countries with UHC (De Vito *et al.*, 2015). These barriers included differing interpretation and implementation of health policies, language and communication problems, lack of social networks, migrants’ fears, and lack of knowledge about their rights, the healthcare system and healthcare professionals (De Vito *et al.*, 2015; Woodward *et al.*, 2013).

In low-and middle-income countries, there is emerging literature on migrants, refugees, health policies and UHC (Guinto *et al.*, 2015; van Hees *et al.*, 2019; Vijayasingham *et al.*, 2019). A 2015 study on the inclusion of migrants in the UHC systems of Indonesia, Malaysia, Philippines, Singapore and Thailand found variations in healthcare coverage of migrants and implementation challenges (Guinto *et al.*, 2015). Notwithstanding Thailand’s success in expanding insurance coverage to undocumented migrants, key challenges included unclear policy messages, bureaucratic hurdles, inadequate coordination and the inconsistent practices of frontline implementers (Suphanchaimat *et al.*, 2015).

In Africa, post-colonial African migration is predominantly intraregional, with South Africa and Nigeria as the leading destination countries (Abebe, 2017). The African Union’s draft 2018–2027 migration policy framework for Africa recommends that migrants should have access to national healthcare systems, but the authors underscore the need for further research on the intersection between the vulnerabilities of migrant populations, healthcare access and entitlement to basic health services (African Union, 2017).

South Africa has a two-tier healthcare system, consisting of an under-resourced public health sector that provides care to around 84% of the population, and a well-resourced private health sector that serves around 16% of the population with access to private health insurance (The Presidency, 2019). Notwithstanding numerous transformation efforts since South Africa’s democratic transition in 1994, it remains one of the most unequal countries in the world (The Presidency, 2019). The 2019 report of the Health Market Inquiry (HMI) provides evidence of the inequities in resource availability and healthcare provision between the public and private health sectors, and the need for major reforms (Competition Commission of South Africa, 2019). Similarly, the 2019 report of the South African Lancet National Commission provides a detailed diagnosis of quality of care gaps and emphasizes the need for, and the transformation potential of, quality UHC in the country (South African Lancet National Commission, 2019). Both the HMI and Lancet National Commission reports underscore the necessity of UHC reforms enunciated in the National Development Plan (NDP) (National Planning Commission, 2011). The proposed National Health Insurance (NHI) system aims to give effect to the progressive realization of the right to healthcare enshrined in the South African Constitution (Republic of South Africa, 1996) and the UHC goals of the NDP (The Presidency, 2012). The proposed NHI system is a major financing reform aimed at overcoming the public–private health sector inequities and moving closer towards quality UHC (NDOH, 2017). NHI implementation is envisaged in various phases over a period of 14 years, with the draft NHI Bill released in 2019 to formalize the regulatory aspects of implementation (NDOH, 2019).

In the public sector, the nine provincial health departments are responsible primarily for health service provision, through a network of hospitals, health centres and primary healthcare clinics (The Presidency, 2019). Although the South African Constitution guarantees the right of access to healthcare services for everyone in the country (Republic of South Africa, 1996), in practice healthcare access for migrants and refugees in the public sector is intertwined with various laws, policies, migrants’ socio-economic and legal status in the country and the behaviours and practices of government officials. Several researchers have highlighted the problems experienced by all categories of international migrants. These include the disjuncture between immigration and health legislation, the lack of or delays in obtaining official documents from the Department of Home Affairs, and the attitudes or actions of frontline immigration and/health workers. These problems impact on migrants’ healthcare access, their experiences, and the ability of health providers to honour their ethical and professional obligations (Alfaro-Velcamp, 2017; Vearey *et al.*, 2018; Crush and Tawodzera, 2014; Zihindula *et al.*, 2015; Hunter-Adams and Rother, 2017). A 2020 published study that examined healthcare providers’ perspectives on migrants in the public sector of Gauteng province found that the majority of these providers disagreed with the inclusion of migrants and refugees in the proposed NHI system and were of the opinion that migrants should return to their home countries for healthcare (White *et al.*, 2020a). Studies have also documented the deleterious experiences of migrants and refugees in the public health sector, characterized by inadequate access to essential treatment, medical xenophobia, discrimination and language barriers (Hunter-Adams and Rother, 2017; Zihindula *et al.*, 2017; Faturiyele *et al.*, 2018). White and colleagues found that migrant patients’ satisfaction with health workers in the public sector were influenced by the receipt of information about their condition: perception of polite treatment, the time spent in facility, and whether they received their prescribed medicines (White *et al.*, 2020b).

Set against the backdrop of South Africa’s health sector reforms, notably the intended implementation of the NHI, and South Africa’s strategic position in Africa, the aim of this study was to explore whether legislation, health policies and the perspectives or actions of health policy actors facilitate UHC for migrants or exacerbate exclusion, and reproduce inequalities and discrimination against migrants. This is part of a larger doctoral study on the experiences of international migrants, social exclusion and health system responsiveness in the public health sector of a South African Province.

## Methods

### Conceptual framework

In this study, we have adapted the conceptual framework of the Social Exclusion Knowledge Network (SEKN) (Popay *et al.*, 2008) to explore legislation, policies and the perspectives of health policy actors on migrants and UHC in South Africa.

Set up as part of the WHO’s Commission on Social Determinants of Health, the SEKN highlighted the contested nature of the concept of social exclusion and the nuances in the discourses on social exclusion in different geographical regions (Popay *et al.*, 2008). The SEKN aimed to develop a definition of social exclusion with global relevance (Popay *et al.*, 2008). Consequently, the SEKN defined social exclusion as a relational concept in recognition of the interdependence of social systems and the centrality of individual and collective action in pursuing policies or actions to promote inclusive and cohesive social systems (Popay *et al.*, 2008). The SEKN defined social exclusion as follows:

*Dynamic, multi-dimensional processes driven by unequal power relationships that operate along and interact across cultural, economic, political and social dimensions and at different levels. Exclusionary processes contribute to health inequalities by creating a continuum of inclusion/exclusion. This continuum is characterised by an unjust distribution of resources and unequal capabilities and rights required to createthe conditions necessary for entire populations to meet and exceed basic needs; enable participatory and cohesive social systems; value diversity; guarantee peace and human rights; and sustain environmental systems* (Popay *et al.*, 2008, p. 36).

**Figure 1.**
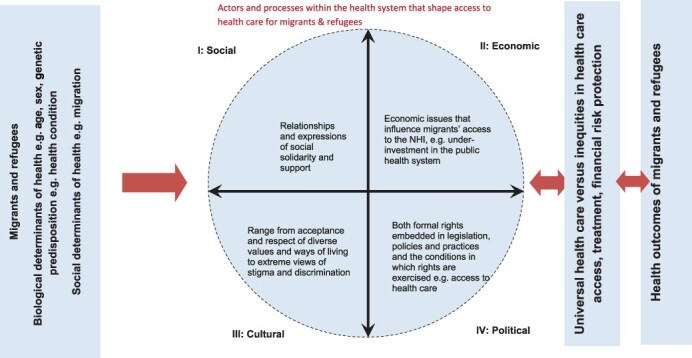
A social exclusion relational framework on migrants, refugees and the UHC in South Africa.

Source: Adapted from Popay *et al.* (2008).

In concert with the relational perspective of social exclusion, our study views the health system in South Africa as a social system, with numerous actors and processes that influence UHC for migrants (Figure 1).

The original SEKN framework assumed that social exclusion processes operate in the context of pre-determined biological (e.g. age) and genetic determinants (Popay *et al.*, 2008). We have added to this context, migration as a social determinant of health (Figure 1), in recognition of its influence on both UHC, and ultimately health outcomes of migrants and refugees (Castaneda *et al.*, 2015).

The original SEKN framework describes four dimensions of overlapping and interconnected power relationships that constitute the continuum from inclusion to exclusion—economic, political, social and cultural (Popay *et al.*, 2008). We kept these four dimensions (Figure 1), but adapted the definitions, as these were too broad for the study purpose. In our study, the social dimension refers to relationships and views of social solidarity and support expressed for the inclusion of migrants and refugees in South Africa’s NHI system, its vehicle towards UHC. The economic dimension considers issues such as access to income and livelihoods that influence migrants’ access to the NHI. The cultural dimension focuses on the perspectives of policy actors on the acceptance and respect of diverse values and ways of living, including extreme views of stigma and discrimination. The political dimension includes the formal rights embedded in legislation, policies and practices and the conditions in which rights are exercised e.g. access to healthcare.

In our adaptation of the SEKN framework, we replaced differential exposure and vulnerabilities with UHC (Figure 1). We suggest that there is a two-way relationship between social exclusion processes and the behaviour and actions of actors and UHC for migrants and refugees. Put differently, UHC (which includes legislation and policies to mediate the actions of policy actors) could reduce social exclusionary processes, whereas inequities in healthcare access, quality treatment and financial risk protection (the essence of UHC) will exacerbate social exclusion. We replaced health inequalities with health outcomes. Drawing on WHO definition of UHC (WHO, 2019), we propose that there is a two-way relationship between UHC and the health outcomes of migrants and refugees. The lack of or exclusion from UHC will exacerbate health inequities and contribute to poor health outcomes. In contrast, UHC will ensure responsiveness to population health needs and achieve more equitable and optimal population health outcomes.

The drawbacks of the SEKN framework are that the definitions are broad, lacking specific criteria to measure social exclusion or its dimensions. The framework was also developed primarily to feed into the work of the Commission on Social Determinants of Health, and prior to the UHC discourse. Nonetheless, the SEKN conceptual framework has several analytical advantages. First, it focuses attention on the structural drivers of exclusion (e.g. laws and/or policies), the processes that generate unequal power relationships (how), the policy actors that drive exclusion (who) and the intersection among all of these (Popay *et al.*, 2008). Second, the framework is useful in highlighting a continuum of inclusion and exclusion, which avoids labelling of migrants and refugees as ‘excluded’, yet recognizes the agency of migrants and refugees in contributing to change. Last, the framework also enables the identification of active exclusionary processes (e.g. policies, outright discrimination, etc.) and passive exclusionary processes (e.g. hospital billing systems, lack of knowledge of existing laws or policies) (Popay *et al.*, 2008).

We used the adapted SEKN conceptual framework in the design of the interview schedule, and the thematic analysis of the documents, and the interviews.

### Study design

A qualitative study design was used, which combined in-depth interviews with key informants (KIs) and a review of government legislation and policies on migrants and refugees. Each component is described below.

### Key informants

The purpose of the key informant interviews (KIIs) was to explore the perspectives of key health policy actors on UHC and the NHI for migrants and refugees in South Africa. We developed a map of all the potential stakeholders, targeting knowledgeable individuals able to provide in-depth and rich information on migrants, their healthcare access, perspectives on social exclusion, and UHC and NHI in South Africa (Table 1). Hence, we compiled a list of 27 potential KIs.

**Table 1.** Map of key policy actors

Category	Possible number
Managers/government officials involved in Green Paper on migration or amendments to immigration bill	1
Senior government managers in Health, Presidency, Treasury, etc.	3
Members of NHI Ministerial task team	2
Managers in charge of mid-year population estimates at Statistics South Africa	1
Purposive selection of managers in charge of hospitals, health centres or clinics where health provider and patient surveys were conducted with at least one person from each type of facility	6
Civil society organizations focusing on or involved in: Migration or migrant rights Provision of services to migrants (e.g. legal advice) Health governance structures	4
United Nations Agencies International Organization of Migration High Commission on Refugees	2
Academics/researchers focusing on: Migration Migrants’ rights Health or health services for migrants UHC and NHI	8

Additionally, we used a snowballing technique, by asking KIs who agreed to interviews to recommend other potential KIs for interviews.

Informed by the adapted conceptual framework, we developed a semi-structured interview schedule covering questions on UHC, NHI, healthcare access for migrants and refugees, the drivers, processes and pathways of social exclusion; and policies, programmes and/or actions to address the health needs of migrants and eradicate social exclusion. The interview schedule was piloted with two participants who have similar profiles to the KIs. Based on the feedback and observations during the pilot, no changes were necessary. The information from the pilot interviews was excluded from the analysis.

Potential KIs were contacted via email or phone for interviews. Following verbal agreements, each consenting KI was provided electronically with the study information sheet, consent forms for the interview and digital recording, and the interview schedule. The principal researcher (JAW) scheduled all the interviews at a mutually convenient time at a venue that ensured privacy during the interview. Following written informed consent for both the interview and the recording, the principal researcher conducted the interviews between March 2015 and December 2019. Six interviews were conducted between 2015 and 2017. Interviews for the remainder of KIs (*n* = 12) were conducted in 2018 and 2019. Because of the lapse of time since the analysis of the interviews in 2020, we sent the transcripts to the six KIs who were interviewed between 2015 and 2017 to validate their responses or to make any changes.

Each interview lasted an average of 1 hour, but the length of time varied depending on the responses of the KI. Interviews were recorded digitally with the participants’ consent, and the principal researcher also took detailed field notes.

### Ethical considerations and researcher positionality

The Human Research Ethics Committee (Medical) Ethics of the authors’ institution provided approval for this. All study participants received a detailed information sheet and provided written consent for both the interview and the digital recording. The principal researcher informed each KI of the voluntary nature of the information and their rights as a study participant. The principal researcher ensured both confidentiality and anonymity of the interview data. The written consent forms are in a secure, locked cupboard, while the interview recordings and transcriptions are on a password-protected computer.

Both authors are employed at a large South African university. The first author is a PhD candidate, whose research focuses on migration as a social determinant of health, the mental health needs of migrants and how these intersect with the health system. The senior author was a member of the SEKN, the former head of a provincial health department, former head (dean) of a university school of public health and has extensive experience of health leadership, research and public health activism. Both authors are passionate about health equity and social justice but recognize the importance of data immersion, ethical research conduct and constant self-reflection.

The authors complied with all aspects of the Singapore Declaration on research integrity (World Conference on Research Integrity, 2010).

### Review of legislation and policies

The purpose of the document review was to examine the content of legislation and policy documents to determine whether these laws and/or health policies were enabling or exclusionary. We used the United Nations and African human rights documents as the foundation for the review of the South African laws and policies. We focused on the period between 1994 and 2020, corresponding with the period of democracy. We assumed that these are the primary documents that shaped the policy and regulatory environment on migration, migrants, health and the South African healthcare system.

The document review took place between February and May 2020. We applied the READ approach—a systematic process to guide document analysis—to the document review (Dalglish *et al.*, 2020). The READ approach involves a systematic four step procedure in reviewing documents: (1) ready your materials, (2) extract data, (3) analyse data and (4) distil your findings (Dalglish *et al.*, 2020, p. 3).

Our first step was to ready our materials for the document review by searching for official and legal documents broadly pertaining to migrants, refugees and health, specifically legislation (international and national) and health policies. We identified 24 potential documents for inclusion in the review (Table 2). These documents were accessed through PubMed, Google Scholar and hand searching of the websites of the UN agencies, and the South African government. Following this initial identification of legislation and policies, the principal researcher then embarked on filtering through the documents to include those specifically related to regulations, rules or protocols on access, utilization and health coverage for migrants and refugees (criteria for inclusion).

**Table 2.** List of documents included in review

United Nations Human Rights Foundation documents (*n* = 6)	
1948 Universal declaration of human rights The 1951 Convention Relating to the Status of Refugees and its 1967 Protocol International Covenant on Economic, Social and Cultural Rights, 1966 Convention Governing the Specific Aspects of Refugee Problems in Africa, 1969 International Covenant on Civil and Political Rights, 1976 Protocols I and II to the Geneva Conventions of 1949 relating to the Protection of Victims of International and Non-International Armed Conflicts, 1949	
Organization of African Union/African Union Human Rights or Migration Policies (*n* = 3)	
African (BANJUL) Charter on Human and Peoples’ rights The migration policy framework for Africa, 2006 EX.CL/276 (IX) The revised migration policy framework for Africa and plan of action (2018–2027) -*draft*	
The Constitution of the Republic of South Africa (1996) (*n* = 1)	
Department of Home Affairs promulgated laws,	Department of Health promulgated laws, White Papers or
White Papers or policies (*n* = 7)	policies Refugees Act, 1998 (*n* = 7)
Refugees Act, 1998 Immigration Act, 2002 Immigration Amendment Act 19, 2004 Immigration Amendment Act No. 13, 2002 Refugees Amendment Act No. 33, 2008 Immigration Amendment Act No. 13, 2011 White paper on international migration for South Africa, July 2017	1997 White Paper for the Transformation of the Health System National Health Act, No. 61 of 2003 Green paper on National Health Insurance in South Africa, 12 August 2011 2015 White paper on the National Health Insurance for South Africa 2017 National Health Insurance Policy: Towards Universal Health Coverage (White Paper) National Health Insurance Bill, June 2018 National Health Insurance Bill, July 2019

In the next step, the principal researcher extracted the relevant information from the 24 documents and entered the data into an MS Excel spreadsheet/grid. This process was guided by our adapted conceptual framework and out research question, i.e. whether legislation and policies facilitate UHC or exacerbate exclusion for migrants and refugees in South Africa.

### Analysis

Audio files from the KIIs were transcribed verbatim in preparation for analysis. The principal researcher checked all transcriptions for accuracy and steered a process of both inductive and deductive coding during analysis.

The first step of analysing the KIIs was to apply a thematic framework analysis (Attride-Stirling, 2001), thereby generating an understanding of KIs’ perceptions on migrants, social exclusion, UHC and the NHI. The principal researcher and three other researchers (including the supervisor LR) read and coded inductively three of the interview transcripts independently. These independent codes were entered into a grid and shared with all researchers, after which inter-coder agreement was reached. Codes were then grouped to form broader themes and sub-themes. The principal researcher then analysed the remainder of the interviews using the agreed upon codes and themes.

Second, we used the adapted SEKN conceptual framework and applied deductive analysis to the interview data. This deductive approach categorized the themes generated from the data according to concepts contained in the conceptual framework (Figure 1). The analysis was an iterative process, comparing and interrogating the inductive and deductive codes, and finally combining the process to obtain a comprehensive picture on the interview data.

A thematic analysis approach was applied to the document review (Braun and Clarke, 2006). Using a data extraction grid, information was aggregated from each policy or legal document. A key outcome was to explore the inclusion of migrants and refugees in healthcare legislation and/or policies. The data extraction was conducted by reviewing each document, examining the information considering the social exclusion conceptual framework and then inserting the relevant information into the grid. Consensus was then reached on the themes contained in the grid.

In the final step of the data analysis, the principal researcher used the adapted conceptual framework and a process of immersion and reflection in the triangulation of the data from the interviews and the document review. These steps in the qualitative data analysis for both datasets ensured the rigor of the research data, specifically credibility, transferability, dependability and confirmability (Lincoln and Guba, 1986).

## Results

Eighteen KIs were drawn from five categories: tiers of government, including public hospitals (*n* = 9); academics or researchers (*n* = 5); civil society organizations (*n* = 3) and a United Nations (UN) Organization (*n* = 1).

Five inter-related themes emerged from the qualitative analysis: disjuncture between international treaties or declarations, the South African Constitution, and national legislation or policies; under-investment, overburdened and dysfunctional healthcare system; complexity and/or contradictory nature of migrant access to healthcare; pathways and processes of social exclusion; and migrant stereotypes, suspicions and discrimination. We present the findings from the document review and the interview themes in an integrated manner.

Table 3 shows the themes and sub-themes, and their alignment to the social exclusion conceptual framework.

**Table 3.** Themes, sub-themes & their alignment to the social exclusion conceptual framework

Interviews and document review		Social exclusion conceptual framework: four relational dimensions			
Themes	Sub-themes	Social = Relationships and expressions of social solidarity and support	Economic = Economic issues that influence migrants’ and refugees access to the NH	Cultural = Range from acceptance and respect of diverse values and ways of living to extreme views of stigma and discrimination	Political = Both formal rights embedded in legislation, policies and practices and the conditions in which rights are exercised e.g. access to healthcare
Disjuncture between international treaties or declarations, the South African Constitution, and national legislation and/or policies	SA is signatory to United Nations Conventions/treaties on refugees SA Constitution contains Bill of Rights (Section 27) Provisions/entitlements for migrants in NHI Bill vague and potentially exclusionary Variations in provincial policies, interpretation or implementation of national policies Lack of/unclear policy guidelines on migrants or their rights	X			X
Under-investment, overburdened and dysfunctional public healthcare system	Under-investment in public healthcare system Resource constraints Non-existent or dysfunctional systems Ethical imperative of migrant inclusion	X	X		X
Complexity and/or contradictory nature of migrant access to healthcare	Frontline health workers lack of knowledge on legal obligations Identity documents as barrier to access Misclassifications of migrants and refugees Discretionary healthcare access to migrants Discrimination by front-line health workers Language differences as access barrier	X	X	X	X
Pathways and processes of social exclusion	Societal values or attitudes Social exclusionary behaviours of health policy actors Denial of access or treatment of migrants and refugees Language and exclusion Fear of victimization and discrimination as a barrier to access	X	X	X	X
Migrant stereotypes, suspicions and discrimination	Migrants and the spread of communicable disease Migrants and overuse of South African public healthcare Discrimination on the basis of limited resources Migration detrimental to South African society	X	X	X	X

### Theme 1: disjuncture between international treaties or declarations, the South African constitution, and national legislation/policies

South Africa is a member of the United Nations, a signatory to the 1951 Convention on the Status of Refugees, its 1967 Protocol (United Nations, 1951), the International Covenant on Economic, Social and Cultural Rights (United Nations, 1976b), the International Covenant on Civil and Political Rights (United Nations, 1976a) and Protocols I and II to the Geneva Conventions of 1949 relating to the Protection of Victims of International and Non-International Armed Conflicts (United Nations, 1949). KIs from national government highlighted these country obligations.

The South African Constitution contains the Bill of Rights, applicable to all people in the country, regardless of their nationality or legal status (Republic of South Africa, 1996). Section 27 of the Constitution states that everyone has the right to have access to healthcare services, and no one may be refused emergency medical treatment (Republic of South Africa, 1996). Most KIs acknowledged the enabling nature of the South African Constitution but highlighted a disjuncture between an enabling Constitution and other laws, notably the Immigration Act (Department of Home Affairs, 2002) and the 2019 NHI Bill (National Department of Health, 2019). In line with the Constitution, the National Health Act is explicit that ‘a health care provider, health worker or health establishment may not refuse a person emergency medical treatment’ (National Department of Health, 2003). While the National Health Care Act does not distinguish between users and eligibility for free health services (National Department of Health, 2003), the Immigration Act makes provision for the identification of citizens or ‘foreigners’ and the request for documents prior to service provision, except in an emergency (Department of Home Affairs, 2002).

Moreover, the 2019 NHI Bill contains a clause on healthcare to ‘migrants, refugees and asylum seekers, and illegal foreigners’, but apart from emergency healthcare treatment and basic services, the Bill is vague on the healthcare entitlements of migrants and refugees (National Department of Health, 2019). Several KIs highlighted that the NHI Bill is potentially unconstitutional in that language such as ‘illegal migrants or foreigners’ used in the bill is discriminatory undersection 9 of the Constitution and therefore exclusionary. One of the KIs noted that although migrants are gaining access there is a disjuncture between that access and how they should be treated:

*There appears to be some hospitals and facilities that are allowing or can’t avoid a large number of foreign patients. A large number of those patients are illegal but they’re gaining access [to the health facilities] in the context of a grey policy area. There isn’t a clear, coherent framework in place as to how they should be treated* (key informant #14, academia).

KIs also highlighted a disjuncture, in the interpretation or implementation of national policies or in some cases, outright discriminatory, provincial policies or procedures. The KI from the UN organization reported on the circulation of 2013 ‘guidelines on foreign patients’ in a provincial health department, distributed without the knowledge or approval of the National Department of Health. In line with this another KI who referred to these 2013 guidelines, said no one took ownership of these types of exclusionary health policies.

*These [guidelines] go contrary to the Constitution and many other pledges and documents…One hospital was categorically refusing care to all refugees, asylum seekers…This kind of practice leads to exclusion* (key informant #4, UN)

One KI noted that the disjuncture or confusion arises due to a lack of a cohesive national legal or policy framework on migrants and refugees clearly outlining their entitlements, or rights to healthcare.

### Theme 2: under-investment, overburdened and dysfunctionality of the public health system

Many KIs—inside and outside government—highlighted that the longstanding under-investment in the public health system exacerbates resource constraints. KIs indicated that migrants and refugees are seen as placing further strain on the resources in the public health system.

KIs from government reported that resource constraints were felt acutely in Gauteng, Limpopo and Mpumalanga provinces, and these contribute to healthcare challenges for migrants.

*Those provinces feel severely constrained by a huge influx [of migrants], which is not budgeted for, in terms of resource allocation. This thing [migrants’ healthcare access] is often a battle for scarce resources* (key informant #8, government).

The characterization of the public healthcare system as overburdened and largely dysfunctional was echoed by non-governmental KIs, noting that poor quality care is experienced by all users of the public health system, but the negative experiences of migrants and refugees are compounded because of their status.

*If you’re relying on the public health system, it’s overburdened, it’s overstretched, there’s long waiting times and everyone experiences that challenge…non-nationals experience something quite specific around being foreign [and] manifests in issues around language, around the way people are treated* (key informant #15, academia)

According to two of the KIs from the academy, the perceived dysfunctionality is because mobility (migration), ethics and human rights are not considered explicitly in the public health system coupled with the lack of appropriate decision-making in the face of financial constraints.

*What is the ethical response to [all] people with services and systems that are subject to financial constraints, quite significant constraints?* (key informant #14, academia)

*The other issue, which we need to understand better, is the way in which decisions are made in a situation of limited resources* (key informant #15, academia)

In addition, KIs felt that the lack of information systems, insufficient costs or costing of service provision and ‘obscure’ decision-making contributes paradoxically to the under-investment in the public health system, which in turn contributes to the social exclusion of migrants and refugees.

One of the KIs from civil society stressed that there needs to do an assessment of South Africa’s international and national obligations, inclusive of migrants and the resources needed to meet these obligations. This should then be followed by an assessment of resource availability, and development of strategies towards the progressive realization of these obligations.

*There needs to an assessment of resources and the spending of resources, what resources are available, what resources are spent, as well as what our international and local obligations are, before cutting out a section of the population…. and not just say, can we tack on some services [for migrants]. They [migrants] can’t be the bottom of the log* (key informant #2, civil society organisation).

### Theme 3: access to the public health system is complex and contradictory

Although both the Constitution and the National Health Care Act stipulate the right of individuals to access essential healthcare (Republic of South Africa, 1996; National Department of Health, 2003), KIs pointed to a complex and at times, contradictory set of issues regarding migrants’ healthcare access. They were of the opinion that access barriers were rooted in the failure of healthcare workers to implement legal service obligations to migrants and refugees, either due to a lack of knowledge on the obligations or outright discrimination.

KIs from civil society organizations put the responsibility of clear guidelines on migrants and refugees at the door of the national and/or provincial health authorities. The failure to provide clarity or policy guidelines leaves the interpretation up to frontline staff creating confusion among healthcare workers about the healthcare entitlements of migrants and refugees.

*There is a lack of information on both sides…. Therefore, when they receive someone [migrant], immediately they see an outsider, a foreigner, they automatically tell the person to go back because they do not know how much should they offer that person at which level, with a document or not* (key informant #3, civil society organisation)

Barriers to access are exemplified by the over insistence on proof of identification prior to service. Several KIs noted that an identity document (ID) often determines healthcare access granted to migrants and refugees. The Immigration Act requires staff at healthcare facilities to determine the status of migrants presenting at facilities for healthcare before providing services and to report all undocumented migrants to the Department of Home Affairs (Department of Home Affairs, 2002).

*People go to facilities and they are turned away simply because they didn’t have a [ID] document* (key informant #3, civil society organisation)

*It’s designed, in my view, to target foreign nationals and to exclude them* (key informant #16, academia)

Another access barrier is the misclassification of refugees in terms of the hospital fee-paying system. Clerical staff classify users through a means test prior to the healthcare consultation. Migrants, in contrast to refugees, are expected to pay in terms of the fee schedule but frontline health workers do not understand the difference, hampering access. In some cases, the misclassification and subsequent denial of care is exacerbated by a lack of explanation to migrant patients.

*That person comes with no cent and when they are sent away sometimes you find that they [healthcare worker] did not explain to them [migrant or refugee] to understand that it’s a requirement [the fee]* (key informant #3, civil society organisation)

These problems may be addressed when civil society organizations intervene to ensure the rightful classification of select migrants and refugees.

*Our refugees and asylum seekers [have] been wrongly classified as private patients and have been made to pay just for a consultation close to R5000 [2020 exchange rates∼294 US dollars; R17=1$], which we have contested many times. It shows a fundamental problem in the whole system if we have to challenge individual cases for re-classification* (key informant #1, civil society organisation)

The bureaucratic hurdles that create access barriers and exclude migrants and refugees intersect with rationing of resources and/or discrimination by frontline health workers.

The Bill of Rights in the Constitution states that ‘No person may unfairly discriminate directly or indirectly against anyone…’ (Republic of South Africa, 1996, p. 6). However, some KIs pointed out that health workers are members of the broader South African society, where discriminatory or xenophobic attitudes are prevalent.

*The average South African is xenophobic, [and] does not like foreigners, feels [foreigners] are using resources. They shouldn’t be [in] clinics. These hospitals are just replicating these other sentiments in society* (key informant #13, academia)

Such discrimination goes against health professional codes of ethics and professionalism and results in health workers managing patients badly, as noted by a KI from government.

In addition, language differences may compromise the quality of care provided by healthcare workers and may influence adherence to treatment, keeping appointments or accessing care.

*Then you also have language barriers that affect not only the care seeker, it’s a factor affecting both sides, which hinder quality, access to quality public healthcare* (key informant #4, UNHRC)

### Theme 4: pathways and processes of social exclusion

Social exclusion manifests itself in the discriminatory and xenophobic attitudes and actions of some frontline health workers towards migrants and refugees. KIs from civil society and academia said the recurring incidents of xenophobic violence in South Africa illustrate a broader process of social exclusion. The discrimination is present in the labour markets and access to employment, membership of social groups, and the possession of the identity document [ID].

One KI pointed out that nurses might verbalize the narrative of politicians.

*I found them [nurses] arguing with patients. ‘Why are you here? Do you want special treatment?’ I think that [expressed discrimination] comes across more because nurses have so much more contact [with patients]. They are in that one ward, 12 hours at a time. I find that nurses forget that a patient is a patient. If you look at the statement made by the MEC and the Minister of Health recently [in 2019], when we were criticized for the state of our hospitals. It was justified by them that we’ve got so many people from the outside that we’re treating* (key informant #5, government)

Another informant noted that some health workers do not hide their discriminatory views of migrants.

*I think what is so incredible [for me] is how open people are. [They say] ‘You are a makwerekwere [a derogatory term for a foreigner], what are you doing here, get out of my clinic’* (key informant #13, academia)

These forms of discrimination also extend to treatment for migrants.

*I’ve had cases of people being denied access to treatment, not because they don’t have rights, but because they are not South Africans or did not belong here as many people would say, which is contrary to what the law says. There are cases of people who have been denied admission to beds because they are not South African. There are cases of people who have been treated or given some kind of treatment and told to go when ordinarily they should be admitted. There are people who have been denied [surgical] operations because they are not South African* (key informant #1, civil society organisation)

However, the situation is more complex than denial of access or discriminatory attitudes. Health system constraints contribute to the feeling of frustration among healthcare workers, who may take this frustration out on patients, especially foreign patients. One KI highlighted language differences as a potential trigger accounting for challenging and frustrating interactions

*Maybe you have one nurse that is going to attend 50 or 100 clients a day. They [nurses] are already overburdened. On top of that, someone comes and doesn’t understand the [local] language, it creates a frustration, and the nurse can just say something [derogatory] out of frustration that’s going to affect the [migrant] patient* (key informant #9, government).

Furthermore, language differences may also serve as an identifier of migration status. While the accent of migrants, despite speaking English fluently, can exacerbate exclusionary behaviours by healthcare providers.

*Language is an expression of culture. People are able to distinguish whether you’re a foreigner or a South African by virtue of the language you speak. Language is an important part of social inclusion or social exclusion. Some refugees or immigrants cannot speak English or any South African language and as a result, our health providers marginalize them* (key informant #10, government)

In addition to language as an identifier, fear of victimization and discrimination can act as a barrier to access public healthcare services. Migrants and refugees may perceive being treated unfairly which sets the tone for their experiences at facilities. The knock-on effect is that migrants and refugees will take on the added expense and rather use private health services.

*The refugee might already know that we shouldn’t be going to that facility, which is the nearest one, because we won’t be treated well there* (key informant #7, government)

*They would rather save up money and go to a private GP than take their chances with the abuse they [might] get from the public service* (key informant #13, academia)

### Theme 5: suspicions, stereotypes and discrimination

Some of the KIs highlighted that suspicions and stereotypes about migrants and refugees often overlay with discrimination and xenophobia resulting in healthcare access barriers. These stereotypes include misconceptions that migrants encourage the spread of communicable diseases, that they overuse South African public health services, as well as the detrimental effects of migrants and migration on the South African society.

The views on migrants and the spread of communicable diseases present both a stereotype, and a public health concern. This perpetuates barriers to access and, furthermore, prevents those migrants and refugees with communicable diseases from seeking treatment. The only way we are going to deal with communicable disease control is by including population mobility in our responses (key informant #15, academia)

KIs noted that there is also a perception among healthcare workers that migrants and refugees come to South Africa for maternal healthcare and have many children to qualify for government grants. While children born in South Africa do not automatically qualify for citizenship, the perception still perpetuates barriers to access.

Another narrative that emerged among KIs was the belief among South African society that migrants deplete already limited resources that should be allocated to South Africans. A KI from the academy highlighted that migrants and refugees are also perceived by citizens as detrimental to South African society. These sentiments echo the existing perceptions that migrants are ‘stealing jobs’ meant for South Africans.

*We don’t live in a society where immigration is viewed positively, so people believe the rhetoric that ‘oh they‘re coming to get the services, they’re coming to steal the jobs, they’re coming to get healthcare’* (key informant #15, academia)

## Discussion

In this qualitative study, we explored whether legislation, health policies and the actions of health policy actors facilitate UHC for migrants, or exacerbate exclusion, and reproduce inequalities and discrimination against migrants. We used the WHO definition of UHC that embodies the concepts of equity, quality and financial risk protection (WHO, 2019). Notwithstanding the contestations around the NHI in South Africa (Gilson, 2019; Zondi and Day, 2019), the 2019 NHI Bill is an important indicator for the intentions of the South African government to provide UHC for migrants and refugees.

Our findings suggest that paradoxically, legislation and policies in South Africa both facilitate and exclude healthcare for migrants. Despite a right-based and non-discriminatory Constitution and an enabling National Health Act, the Immigration Act and the 2019 NIH Bill make the legal status of migrants the most significant determinant of healthcare access (Republic of South Africa, 1996; Department of Home Affairs, 2002; National Department of Health, 2003; 2019). This disjuncture is exacerbated by the reported variations in content, interpretation and/or implementation of policies at the provincial level. Abbas *et al.* have highlighted the erratic and volatile nature of policies towards migrants in Europe and the USA that are unresponsive to their needs (Abbas *et al.*, 2018). Sargent has argued that access to healthcare for migrants is a product of [health] policies of entitlement or exclusion, reflecting the notion of ‘deservingness’ or the moral worth of migrants to quality health services (Sargent, 2012). Other studies also illustrate the influence of legal status on migrants’ access to comprehensive health services (Hannigan *et al.*, 2016; Chiarenza *et al.*, 2019). Both WHO and the IOM have stated unequivocally that achieving the SDG target on UHC is dependent on meeting the health needs of migrants and refugees and ensuring that they have access to quality and affordable health services (International Organization for Migration (IOM), 2019). Although there is recognition of the mammoth task in achieving migrant-friendly, inclusive health systems that will ensure UHC, this aspirational goal also provides an opportunity to promote a more coherent and integrated approach to health and well-being, rather than vertical disease-specific interventions (International Organization for Migration (IOM), 2019; Abubakar and Zumla 2018; Abubakar *et al.*, 2018).

There was consensus among KIs regarding the resource constraints and under-investment in the public health sector. These in turn contribute to the perceived dysfunctionality of the public healthcare system, which affects the financial classification, quality of care and access for all patients. Other South African studies have demonstrated the under-investment, fault lines and burden of care in the public health sector (Rispel, 2016; South African Lancet National Commission, 2019; The Presidency, 2019; OXFAM South Africa, 2020). An OXFAM South Africa (2020) research study found a combination of neoliberal economic policies, insufficient investment in the health system and workforce, and poor implementation of existing legislation have created the perfect storm of inequities and fragility of the South African health system. Several initiatives are addressing the major diagnostic problems in South Africa’s health system (The Presidency, 2019). However, most KIs reported the experiences of health system responsiveness were worse for migrant patients, who encountered a combination of structural, social, cultural and economic exclusion. Although the intersection of under-investment, resource constraints and dysfunctionality contribute to the exclusionary behaviour and actions of frontline health workers, this cannot condone the reported discrimination and stigma experienced by migrant patients.

In our study, the reported discrimination and stigma experienced by migrants included the request for IDs, financial misclassification, discretionary healthcare access, denial of treatment and sometimes name-calling and outright discrimination by frontline health workers. These are examples of what Crush and Tawodzera have termed ‘medical xenophobia’, or the ‘negative attitudes and practices of health sector professionals and employees towards migrants and refugees on the job’ (Crush and Tawodzera, 2011, p. 655). KIs from government pointed out that frontline health workers reflect the negative societal stereotypes and express political sentiments about migrants and migration. These negative stereotypes were also found in a 2017 study that examined post-apartheid narratives about ‘foreigners’, with African migrants blamed for all social ills in the country (Pineteh, 2017). Pineteh (2017) found African migrants are framed as the adversary blamed for all social ills in the country. White and colleagues found that 21.0% of the 277 health workers surveyed in public health facilities of a South African province reported that they had witnessed discrimination against migrants, while 22.6% reported differential treatment of migrant patients (White *et al.*, 2020a). Surprisingly, KIs from government did not condemn these incidents of discrimination that they relayed, even if the culprits did not represent the views of the majority of health workers or the South African public health system.

The review of migrants’ access to healthcare services in the EU does not mention discrimination explicitly (Hannigan *et al.*, 2016). However, experiences from Thailand have shown the need for high-level political leadership to protect the human rights of migrants and principles of non-discrimination in accessing health services (Suphanchaimat *et al.*, 2016).

Our study findings suggest that civil society organizations played an important role, intervening and mediating access to care for migrants. Other studies have highlighted the role of civil society in advocating for the health rights of migrants (Ambrosini and Van der Leun, 2015; Orcutt *et al.*, 2020), or in ensuring quality UHC (Brolan *et al.*, 2017).

There are a few recommendations that arise from this study. First, evidence suggests that the removal of legal restrictions is an important prerequisite for UHC, even though it does not guarantee equitable, quality healthcare access for migrants (Abbas *et al.*, 2018; Legido-Quigley *et al.*, 2019). Although Germany is a high-income country with a different context to South Africa, a study that examined the effects of restricted access and two major policy reforms on incident health expenditures for asylum seekers and refugees found the cost of exclusion was much higher (Bozorgmehr and Razum, 2015). This led to both delayed care, and increased costs of care (Bozorgmehr and Razum, 2015). In South Africa, the NHI Bill both restricts healthcare access for migrants and appears to be unconstitutional. Several individuals and civil society organizations previously made submissions or inputs on the NHI and migrant exclusion. Our study findings also suggest the need for revisions to the NHI Bill to ensure at the health system is responsive to the needs of migrants and refugees. Such revisions would also be in line with the South African Constitution, and the draft migration policy framework of the African Union that recommends UHC for all migrants (African Union, 2017). In addition, revisions to the NHI Bill will ensure that the government meets its international obligations in respecting and promoting the human rights of migrants and refugees (International Labour Office (ILO) *et al.*, 2001; International Organization for Migration (IOM), 2019).

Several scholars have shown that legislation on its own cannot bring about effective change (Adjai and Lazaridis, 2014; Alfaro-Velcamp, 2017; Legido-Quigley *et al.*, 2019; OXFAM South Africa, 2020). Hence, our second recommendation relates to the importance of addressing the resource constraints and under-investment in the South African public healthcare system. Although improving the performance of the health system is a key government priority (The Presidency, 2019), leadership and implementation remain problematic. Investment in health and in the health system will facilitate quality UHC for all, including for migrants and refugees (South African Lancet National Commission, 2019; Oxfam South Africa, 2020).

The KIs in our study reported various incidents of medical xenophobia, which require a conscious and comprehensive effort to address. Scholars have argued that the pervasive xenophobia in South Africa requires multi-prong strategies at individual-, organizational- and state-level strategies (Adjai and Lazaridis, 2014; Tella, 2016). Our third recommendation relates to the responsibility of senior public servants and health managers to ensure health professionals uphold their professional codes of conduct. Health professionals are bound by codes or ethics, and they pledge to practice with conscience, dignity and without discrimination and to advocate on behalf of vulnerable and disadvantaged patients, regardless of gender, political persuasion and nationality (Gallagher and Little, 2017; Meier *et al.*, 2018; Amon and Friedman, 2020). Drawing on the Rollback Xenophobia campaign, a broad coalition of UN, government, civil society organizations, health professions councils and representative organizations is needed to prevent and combat medical xenophobia (National Consortium on Refugee Affairs (NCRA) *et al.,* 1998). Such a campaign should focus on human rights, zero tolerance towards xenophobia and other types of discrimination, mutual respect and the importance of Pan-Africanism (Tella, 2016). The campaign should be complemented by continuing professional development programmes on human rights, ethical dilemmas in the health system and its responsiveness. Furthermore, user-friendly clear guidelines should be developed that include the rights and responsibilities of healthcare providers, migrants and refugees, and healthcare entitlements. The coalition should ensure the media plays a role in condemning xenophobic and discriminatory attitudes and actions.

The relatively small number of KIs limited our study. KIs from civil society organizations were primarily Gauteng based, and only one was from a UN organization. Hence, the findings may not be representative of other provinces in South Africa. However, we reached data saturation after 10 interviews. The findings provide a glimpse of the experiences of migrants in the public health sector, as educated migrants in higher socioeconomic groups are more likely to have private health insurance and would not utilize the public health sector. Although six KIs confirmed their views in 2020, the interviews were conducted over a 5-year period, and this is a potential limitation.

Nonetheless, our study has several strengths. First, the SEKN social exclusion model is a useful, analytical framework to explore whether policies facilitate UHC or exacerbate exclusion for migrants. A methodological strength is the combination of KIIs and a document review to explore whether healthcare policies facilitate UHC or exacerbate exclusion for migrants in South Africa. We obtained rich narratives on UHC and migrants in South Africa, which adds to the discourse on a migrant-friendly health system in South Africa as well as the global discourse on UHC for migrants and refugees (Vearey *et al.*, 2017).

Future research should explore the evolution of the NHI legislation and policy development on migrants and refugees using the health component of the IOM’s Migration Integration Policy Index (Abbas *et al.*, 2018). The health indicators cover four dimensions: entitlement to health services, policies to facilitate healthcare access, responsive health services and measures to achieve change (Abbas *et al.*, 2018). Another potential research area could be a comparative analysis of legislation and policies on migrants and UHC in South Africa and other countries with a similar level of income. Such research could also explore the reasons for the contradictions in legislation, policies and actual implementation, as well as the actors, processes and power dynamics that contribute to these contradictions. The important role that civil society plays in mediating access for migrants in South Africa should be also be investigated. Last, further research could compare the experiences of migrants and refugees with those of other vulnerable groups, whether by geography (e.g. rural, informal settlements), health condition (HIV, disability) and/or sexual preferences (LGBTIQ+).

## Conclusion

Health legislation and policies shape healthcare coverage and ultimately, access to and utilization of healthcare services for migrants in South Africa. Our study highlighted the contradictions that exist between an enabling Constitution and National Health Care Act and an exclusionary Immigration Act and NHI Bill. Our study underscores the need for revisions to the NHI Bill to ensure an inclusive healthcare system for all people, regardless of nationality. However, legislative changes need to be accompanied by investment in the South African health system, strategies to improve its performance, value-based leadership and management of the health system, and a broad coalition that ensures the prevention and mitigation of medical xenophobia.

## Data Availability

The data underlying this article will be shared on reasonable request to the corresponding author.
